# The association between inflammatory indices and acute pancreatitis severity: a retrospective cohort study

**DOI:** 10.3389/fsurg.2026.1764029

**Published:** 2026-02-26

**Authors:** Huicong Ma, Na Li, Huaisheng Zhang, Zepeng Shen, Jie Yang, Qiaojie Bi, Xiaoxiao Miao

**Affiliations:** 1 Institute of Emergency and Critical Care Medicine, Qingdao Hospital, University of Health and Rehabilitation Sciences (Qingdao Municipal Hospital), Qingdao, Shandong, China; 2 Department of Emergency Surgery, The First Affiliated Hospital of Bengbu Medical University, Bengbu, Anhui, China; 3 Qingdao Traditional Chinese Medicine Hospital, Qingdao Hiser Hospital Affiliated with Qingdao University, Qingdao, Shandong, China

**Keywords:** acute pancreatitis, biomarker, C-reactive protein-to-calcium ratio, inflammatory biomarkers, severity prediction, threshold effect

## Abstract

**Background:**

Acute pancreatitis (AP) is a heterogeneous inflammatory disease, with ∼20% of patients progressing to moderate-to-severe (MSAP) or severe AP (SAP), conditions associated with high mortality. Early risk stratification is therefore critical. This study systematically evaluated and compared 12 inflammatory biomarkers for predicting AP severity.

**Methods:**

This retrospective cohort included 1,981 hospitalized AP patients (January 2018-December 2023). According to the revised Atlanta criteria, patients were classified into mild AP (MAP, *n* = 1,058) and MSAP/SAP (*n* = 923) groups. Twelve inflammatory indices—monocyte-to-lymphocyte ratio (MLR), lymphocyte-to-monocyte ratio (LMR), C-reactive protein-to-albumin ratio (CAR), C-reactive protein-albumin-lymphocyte index (CALLY), C-reactive protein-to-calcium ratio (CCR), C-reactive protein-to-lymphocyte ratio (CLR), red cell distribution width-to-albumin ratio (RDW/Alb), neutrophil-to-albumin ratio (NAR), systemic inflammatory response index (SIRI), neutrophil-to-lymphocyte ratio (NLR), platelet-to-lymphocyte ratio (PLR), and systemic immune-inflammation index (SII)—were calculated. A multivariate logistic regression model adjusted for 28 covariates. ROC curves assessed predictive performance; restricted cubic splines (RCS) explored nonlinear relationships; and threshold effect analysis was conducted for the highest-performing biomarker.

**Results:**

In the fully adjusted model, nine biomarkers were significantly associated with MSAP/SAP risk: MLR (OR = 1.29, 95%CI: 1.15–1.45), LMR (OR = 0.75, 95%CI: 0.66–0.85), CAR (OR = 3.82, 95%CI: 3.18–4.64), CALLY (OR = 0.56, 95%CI: 0.49–0.64), CCR (OR = 4.84, 95%CI: 3.98–5.96), CLR (OR = 2.12, 95%CI: 1.84–2.46), RDW/Alb (OR = 1.74, 95%CI: 1.54–1.99), NAR (OR = 1.44, 95%CI: 1.27–1.64), and SIRI (OR = 1.29, 95%CI: 1.15–1.46). CCR demonstrated the highest observed accuracy (AUC = 0.768, 95%CI: 0.737–0.799). Threshold effect analysis revealed a nonlinear association, with an inflection point at 15: no significant association was observed below this threshold (OR = 1.015, *P* = 0.558), whereas risk significantly increased above it (OR = 1.212, *P* < 0.001).

**Conclusion:**

Among 12 inflammatory biomarkers, CCR showed the strongest predictive value for MSAP/SAP, with a critical threshold of 15. As an easily obtainable marker, CCR may serve as a practical early warning tool to guide clinical management and risk stratification in AP.

## Introduction

Acute pancreatitis (AP) is an inflammatory disease caused by abnormal activation of pancreatic enzymes, leading to damage of the pancreas, adjacent tissues, and other organs (1). The global incidence of AP is approximately 34 cases per 100,000 persons per year and continues to rise (2, 3). The primary etiologies are gallstones and alcohol consumption, although other causes include hypertriglyceridemia, autoimmune diseases, trauma, and genetic predisposition (4, 5). The clinical course of AP varies considerably: about 80% of patients experience mild disease, while the remaining 20% develop severe acute pancreatitis (SAP), which may lead to peritonitis, pancreatic necrosis, and multiple organ dysfunction, with mortality rates reaching 20%–40% (6–8). Early and accurate identification of patients at high risk of progressing to SAP, followed by timely intervention, is therefore essential to reduce complications and improve clinical outcomes.

Several scoring systems have been developed to assess AP severity, including the Ranson score, Acute Physiology and Chronic Health Evaluation II (APACHE II) score, Bedside Index for Severity in Acute Pancreatitis (BISAP), and Computed Tomography Severity Index (CTSI) (9). Although widely used, each has limitations that restrict their clinical applicability (10).

In SAP, excessive inflammatory mediator release activates an inflammatory cascade, which promotes bacterial translocation and secondary injury to distant organs (11). Previous studies have examined associations between inflammatory indices and AP severity, including C-reactive protein-to-lymphocyte ratio (CLR) (11), neutrophil-to-lymphocyte ratio (NLR) (12), C-reactive protein-to-calcium ratio (CCR) (13), systemic immune-inflammation index (SII) (14), systemic inflammation response index (SIRI) (12), monocyte-to-lymphocyte ratio (MLR) (12), platelet-to-lymphocyte ratio (PLR) (15), C-reactive protein (CRP) (16), lymphocyte-to-monocyte ratio (LMR) (17), and C-reactive protein-to-albumin ratio (CAR) (18). However, prior evidence has been inconsistent, often lacking comprehensive comparisons, and studies in Asian populations remain limited. For example, Tanoğlu et al. reported that NLR may be unreliable in predicting AP severity due to confounding factors such as comorbid diseases (19), while Liu et al. demonstrated that SII had predictive potential, whereas NLR and PLR showed higher specificity and sensitivity (20).

Given these inconsistencies, relatively small sample sizes, and limited direct comparison with CRP in existing studies, further investigation is warranted.

We therefore conducted a large-scale retrospective cohort study to systematically evaluate and compare the predictive value of 12 inflammatory indices (CLR, NLR, CCR, SII, SIRI, MLR, PLR, CRP, LMR, CAR, C-reactive protein-albumin-lymphocyte index [CALLY], and red cell distribution width-to-albumin ratio [RDW/Alb]) in determining AP severity and to identify the optimal prognostic biomarker.

## Method

### Data sources and study population

This hospital-based retrospective cohort study included patients admitted with acute pancreatitis between January 2018 and December 2023. Data were extracted from the hospital's electronic medical record system. Time zero was defined as the first qualifying hospital admission for acute pancreatitis during the study period. The prediction horizon was defined as the occurrence of moderately severe or severe acute pancreatitis (MSAP/SAP) during the same index hospitalization, in accordance with the Revised Atlanta Classification.

The following demographic and clinical variables were collected: sex, age, body mass index (BMI), waist circumference, body temperature, heart rate, respiratory rate, systolic blood pressure, diastolic blood pressure, history of hypertension, diabetes, fatty liver, hyperlipidemia, alcohol use, smoking, etiology (biliary, hyperlipidemia, alcohol-related, unknown), complete blood count parameters, liver and renal function tests, lipid profile, pancreatic enzymes, CRP, procalcitonin (PCT), heparin-binding protein (HBP), lactate, and coagulation indices. Laboratory parameters used in the primary analyses (including CRP, serum calcium, complete blood count, albumin, PCT, HBP, lactate, and coagulation indices) were obtained from the first blood sample collected within 24 h of hospital admission, prior to the development of persistent organ failure.

### Inclusion and exclusion criteria

**Inclusion:** all patients hospitalized with AP from January 2018 to December 2023.

**Exclusion:** (1) chronic pancreatitis; (2) multiple malignancies (pancreatic, esophageal, colorectal, breast, etc.); (3) pregnancy; (4) incomplete medical records; (5) age <18 or >80 years; (6) admission >7 days after symptom onset.

**Figure 1:**
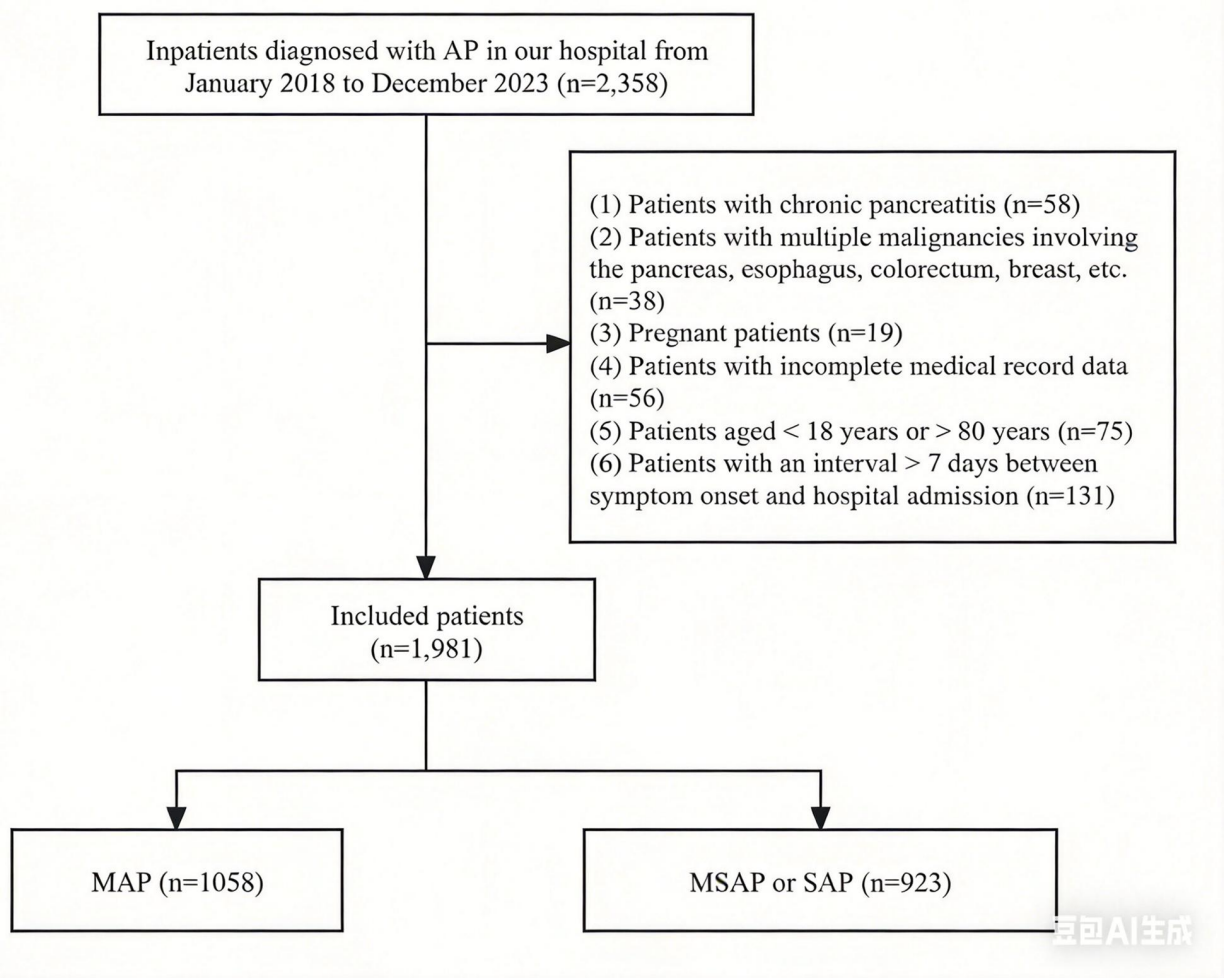
Flow chart of study population selection. MAP, mild acute pancreatitis; MSAP, moderately severe acute pancreatitis; SAP, severe acute pancreatitis.

A total of 1,981 patients were included. According to the revised Atlanta classification [20], patients were categorized into mild AP (MAP, *n* = 1,058) and moderately severe or severe AP (MSAP/SAP, *n* = 923). The study flowchart and patient selection process are shown in Figure 1. MAP is defined as the absence of organ failure and complications; MSAP is characterized by transient (<48 h) organ failure or local/systemic complications without persistent organ failure; SAP is defined as persistent (≥48 h) single or multiple organ failure.

### Sample size calculation

This study was designed as an etiologic association analysis rather than a predictive modeling study. Therefore, using the “10 events per variable” principle (21), the 923 MSAP/SAP cases exceeded the required minimum of 280, confirming adequate sample size.

### Ethical considerations

The study was conducted in compliance with the Declaration of Helsinki (22) and approved by the Ethics Committee of the First Affiliated Hospital of Bengbu Medical College (Approval No.: 2020KY073). As anonymized retrospective data were used, informed consent was waived (23). Patient confidentiality was maintained through encryption and strict privacy protocols.

### Inflammatory Index calculation formulas

To assess the relationship between inflammatory status and AP severity, the following inflammatory indices were calculated:

NLR = Neutrophils/Lymphocytes (15);

PLR = Platelets/Lymphocytes (15);

MLR = Monocytes/Lymphocytes (24);

LMR = Lymphocytes/Monocytes (25);

CAR = CRP/Albumin (26);

CALLY = CRP/(Albumin × Lymphocytes) (27);

CCR = CRP/Calcium (13);

CLR = CRP/Lymphocytes (28),

RDW/Alb = RDW/Albumin (25),

SII = Platelets × Neutrophils/Lymphocytes (20),

NAR = Neutrophils/Albumin (29),

SIRI = Neutrophils × Monocytes/Lymphocytes (29),

### Statistical analysis

Baseline patient characteristics were described according to AP severity (MAP vs. MSAP/SAP). Continuous variables were expressed as mean ± standard deviation for normally distributed data or as median (interquartile range) for non-normally distributed data, while categorical variables were expressed as frequencies and percentages. Group differences were assessed using ANOVA for normally distributed continuous variables, the Kruskal–Wallis test for non-normally distributed continuous variables, and the chi-square test for categorical variables. For comparability of effect estimates across predictors with different measurement scales, continuous variables included in the logistic regression models were standardized using z-score transformation (mean = 0, standard deviation = 1). Accordingly, odds ratios (ORs) derived from logistic regression analyses represent the change in odds per one–standard deviation increase in the corresponding standardized predictor.

To evaluate the association between the 12 inflammatory indices and the incidence of MSAP/SAP, univariate and multivariate logistic regression models were used to calculate odds ratios (ORs) and 95% confidence intervals (CIs). Model 1 was unadjusted, Model 2 was adjusted for gender and age, and Model 3 was further adjusted for the following covariates: gender, age, BMI, waist circumference, body temperature, heart rate, respiratory rate, systolic blood pressure, diastolic blood pressure, history of hypertension, diabetes, fatty liver, hyperlipidemia, alcohol consumption, smoking, etiology, hematocrit, platelet count, creatinine, BUN, sodium, potassium, chloride, PCT, HBP, lactate, PT, APTT, TT, and INR, to assess the robustness of the associations after extensive adjustment, however, the possibility of overadjustment cannot be completely excluded. VIF analysis was performed to test for multicollinearity. Restricted cubic spline (RCS) analysis was applied to explore nonlinear associations between inflammatory indices and MSAP/SAP incidence. RCS models were fitted using four knots placed at the 5th, 35th, 65th, and 95th percentiles of each marker distribution, following Harrell's recommended default settings. Standardization was not applied to CCR in analyses involving nonlinear relationships or absolute cutoff determination. ROC curve analysis was conducted to evaluate the discrimination performance of each inflammatory index. For the index with the greater discrimination performance, a segmented logistic regression model was applied to identify potential threshold effects, in which the change point was estimated using an iterative algorithm to determine the optimal cutoff value. To assess the robustness of the discrimination performance and account for potential optimism, internal validation was performed using bootstrap resampling with 1,000 iterations. Optimism-corrected area under the receiver operating characteristic curve (AUC) was calculated for each index. Differences in AUCs between inflammatory markers were formally compared using the DeLong test for correlated ROC curves, with corresponding *P* values reported.

All statistical analyses were performed using R software (version 4.4.1, R Foundation, http://www.R-project.org), and statistical significance was defined as a two-sided *P* < 0.05.

## Results

### Baseline characteristics

This study included patients with MAP (*n* = 1,058) and MSAP/SAP (*n* = 923), and baseline characteristics are summarized in Table 1. Compared to the MAP group, the MSAP/SAP group exhibited significantly higher values for the following indicators: age, BMI, waist circumference, heart rate, respiratory rate, prevalence of diabetes, fatty liver, history of hyperlipidemia, history of alcohol consumption, history of smoking, white blood cell count, red blood cell count, neutrophil count, monocyte count, hemoglobin, hematocrit, RDW, triglycerides, CRP, PCT, HBP, PT, INR, fibrinogen, NLR, PLR, MLR, CAR, CCR, CLR, RDW/Alb, SII, NAR, and SIRI.

**Table 1:** Baseline characteristics of patients with acute pancreatitis stratified by severity.

Variable	MAP (*N* = 1,058)	MSAP/SAP (*N* = 923)	*P value*
Age (years)	49.00 (38.00, 63.00)	51.00 (39.00, 66.00)	0.015
Gender			0.032
Female	440 (42%)	429 (46%)	
Male	618 (58%)	494 (54%)	
BMI (kg/m^2^)	25.40 (22.40, 28.70)	26.20 (23.60, 29.30)	<0.001
Waist circumference (cm)	87.40 (81.50, 93.90)	89.80 (83.80, 95.70)	<0.001
Temperature ( °C)	37.70 (36.40, 39.00)	37.60 (36.20, 39.00)	0.259
Heart rate (bpm)	88.00 (78.00, 102.00)	98.00 (83.00, 112.00)	<0.001
Respiratory rate (bpm)	21.00 (20.00, 22.00)	22.00 (20.00, 24.00)	<0.001
Systolic BP (mmHg)	132.00 (118.00, 145.00)	131.00 (117.00, 145.00)	0.397
Diastolic BP (mmHg)	82.50 (74.00, 93.00)	81.00 (73.00, 91.00)	0.072
Hypertension	217 (21%)	148 (16%)	0.012
Diabetes	249 (24%)	415 (45%)	<0.001
Fatty liver	612 (58%)	712 (77%)	<0.001
Hyperlipemia history	402 (38%)	657 (71%)	<0.001
Drinking	438 (41%)	515 (56%)	<0.001
Smoking	333 (31%)	413 (45%)	<0.001
Etiology			0.106
Biliary	455 (43%)	366 (40%)	
Hyperlipemia	58 (5%)	57 (6%)	
Alcohol abuse	316 (30%)	319 (35%)	
Unknown	229 (22%)	181 (20%)	
WBC count (×10⁹/L)	10.19 (7.35, 14.21)	12.46 (8.96, 16.63)	<0.001
RBC count (×10^12^/L)	4.56 (4.28, 4.86)	4.90 (4.56, 5.17)	<0.001
Neutrophil count (×10⁹/L)	10.22 (6.95, 13.82)	11.80 (8.26, 15.66)	<0.001
Lymphocyte count (×10⁹/L)	1.16 (0.81, 1.64)	1.11 (0.78, 1.46)	0.005
Monocyte count (×10⁹/L)	0.62 (0.41, 0.90)	0.76 (0.51, 1.03)	<0.001
Platelet count (×10⁹/L)	203.00 (161.00, 249.00)	205.00 (161.00, 253.00)	0.753
Hemoglobin (g/L)	142.00 (132.00, 153.00)	154.00 (142.00, 165.00)	<0.001
Hematocrit	0.42 (0.39, 0.45)	0.44 (0.41, 0.48)	<0.001
RDW (%)	13.60 (12.90, 14.40)	13.80 (13.20, 14.60)	<0.001
ALT (U/L)	28.00 (13.00, 61.00)	29.00 (13.00, 63.00)	0.832
AST (U/L)	27.00 (15.00, 49.00)	26.00 (13.00, 48.00)	0.130
ALP (U/L)	81.00 (61.00, 112.00)	65.00 (52.00, 78.00)	<0.001
Total bilirubin (*μ*mol/L)	19.60 (14.00, 28.30)	19.60 (13.40, 28.10)	0.452
Albumin (g/L)	41.80 (37.70, 45.30)	37.90 (33.20, 42.60)	<0.001
Globulin (g/L)	34.00 (30.20, 38.40)	34.00 (29.90, 38.40)	0.547
Creatinine (μmol/L)	67.00 (60.00, 74.00)	67.00 (59.00, 74.00)	0.587
BUN (mmol/L)	3.97 (3.08, 5.04)	3.95 (3.03, 5.08)	0.764
Sodium (mmol/L)	140.75 (135.30, 145.70)	139.70 (133.60, 144.00)	<0.001
Potassium (mmol/L)	4.00 (3.85, 4.18)	4.00 (3.84, 4.19)	0.988
Chloride (mmol/L)	105.70 (101.80, 109.50)	106.00 (101.80, 109.70)	0.627
Calcium (mmol/L)	2.14 (2.05, 2.25)	1.91 (1.78, 2.05)	<0.001
Glucose (mmol/L)	7.27 (5.97, 9.70)	8.12 (6.41, 10.91)	<0.001
Total cholesterol (mmol/L)	5.13 (3.57, 7.21)	5.27 (3.17, 8.35)	0.490
Triglycerides (mmol/L)	1.66 (1.05, 3.40)	1.79 (1.11, 3.96)	0.020
HDL-C (mmol/L)	1.14 (0.92, 1.34)	1.01 (0.71, 1.26)	<0.001
LDL-C (mmol/L)	2.93 (2.27, 3.76)	3.03 (2.34, 3.89)	0.096
Lipase (U/L)	1,084.50 (664.00, 2,486.00)	1,126.00 (677.00, 2,511.00)	0.333
Serum amylase (U/L)	249.00 (86.00, 818.00)	282.00 (92.00, 778.00)	0.468
Urine amylase (U/L)	1,067.00 (334.00, 4,857.00)	1,071.00 (306.00, 4,408.00)	0.343
CRP (mg/L)	26.91 (21.40, 35.62)	39.76 (27.65, 68.82)	<0.001
PCT (ng/mL)	0.24 (0.11, 0.98)	0.37 (0.13, 1.25)	<0.001
HBP (pg/mL)	38.85 (22.40, 74.10)	47.10 (28.20, 78.70)	<0.001
Lactate (mmol/L)	1.37 (0.96, 2.32)	1.44 (0.98, 2.36)	0.320
PT (sec)	14.30 (13.70, 15.00)	14.50 (13.80, 15.20)	<0.001
APTT (sec)	37.80 (35.90, 39.80)	37.80 (36.00, 39.90)	0.659
TT (sec)	16.70 (16.00, 17.40)	16.70 (15.90, 17.50)	0.698
INR	1.10 (1.04, 1.17)	1.13 (1.05, 1.19)	<0.001
Fibrinogen (g/L)	4.86 (3.73, 6.25)	5.88 (4.88, 7.12)	<0.001
Neutrophil-to-lymphocyte ratio (NLR)	8.92 (5.13, 14.04)	10.93 (7.21, 16.15)	<0.001
Platelet-to-lymphocyte ratio (PLR)	172.00 (124.55, 246.60)	187.50 (132.14, 255.68)	0.005
Monocyte-to-lymphocyte ratio (MLR)	0.54 (0.33, 0.87)	0.69 (0.42, 1.11)	<0.001
Lymphocyte-to-monocyte ratio (LMR)	1.86 (1.15, 3.04)	1.45 (0.90, 2.37)	<0.001
CRP-to-albumin ratio (CAR)	0.66 (0.52, 0.89)	1.08 (0.67, 1.90)	<0.001
CRP-albumin-lymphocyte index (CALLY)	0.16 (0.11, 0.25)	0.09 (0.05, 0.16)	<0.001
CRP-to-calcium ratio (CCR)	12.65 (10.20, 16.63)	21.08 (14.30, 35.72)	<0.001
CRP-to-lymphocyte ratio (CLR)	24.52 (16.90, 37.53)	40.13 (23.59, 64.16)	<0.001
Red cell distribution width-to-albumin ratio (RDW/Alb)	0.33 (0.29, 0.37)	0.37 (0.32, 0.43)	<0.001
Systemic immune-inflammation index (SII)	1,763.90 (994.56, 2,820.61)	2,159.63 (1,318.88, 3,272.73)	<0.001
Neutrophil-to-albumin ratio (NAR)	0.25 (0.17, 0.33)	0.31 (0.21, 0.42)	<0.001
Systemic inflammation response index (SIRI)	5.26 (2.73, 9.91)	8.09 (4.36, 13.28)	<0.001

MAP, mild acute pancreatitis; MSAP, moderately severe acute pancreatitis; SAP, severe acute pancreatitis.

Conversely, the MSAP/SAP group demonstrated significantly lower values for the following indicators: prevalence of hypertension, ALP, albumin, sodium, calcium, HDL-C, LMR, and CALLY.

### Logistic regression analysis

VIF analysis indicated that none of the covariates exhibited multicollinearity, as all VIF values were less than 4 (Supplementary Table 1). In the logistic regression analysis, three models were constructed sequentially: Model 1 (unadjusted), Model 2 (adjusted for gender and age), and Model 3 (further adjusted for 28 covariates including BMI, waist circumference, vital signs, comorbidities, laboratory indicators, and coagulation function). Model 3 passed collinearity detection with all VIF values less than 5. In Model 3, MLR (OR = 1.29, 95%CI: 1.15–1.45, *P* < 0.001), LMR (OR = 0.75, 95%CI: 0.66–0.85, *P* < 0.001), CAR (OR = 3.82, 95%CI: 3.18–4.64, *P* < 0.001), CALLY (OR = 0.56, 95%CI: 0.49–0.64, *P* < 0.001), CCR (OR = 4.84, 95%CI: 3.98–5.96, *P* < 0.001), CLR (OR = 2.12, 95%CI: 1.84–2.46, *P* < 0.001), RDW/Alb (OR = 1.74, 95%CI: 1.54–1.99, *P* < 0.001), NAR (OR = 1.44, 95%CI: 1.27–1.64, *P* < 0.001), and SIRI (OR = 1.29, 95%CI: 1.15–1.46, *P* < 0.001) were significantly associated with the risk of MSAP/SAP. In contrast, NLR (*P* = 0.11), PLR (*P* = 0.40), and SII (*P* = 0.091) did not show statistical significance after multivariate full adjustment. The associations between inflammatory markers and MSAP/SAP are shown in Table 2.

**Table 2:** Association of inflammatory markers with mild acute pancreatitis and severe acute pancreatitis (MSAP/SAP) using logistic regression models.

Inflammatory Marker	Model 1			Model 2			Model 3		
	OR	95% CI	*P* value	OR	95% CI	*P* value	OR	95% CI	*P* value
NLR	1.26	(1.15, 1.38)	<0.001	1.24	(1.13, 1.36)	<0.001	1.10	(0.98, 1.23)	0.11
PLR	1.12	(1.03, 1.23)	0.011	1.10	(1.01, 1.21)	0.034	1.05	(0.93, 1.19)	0.4
MLR	1.37	(1.25, 1.51)	<0.001	1.35	(1.23, 1.49)	<0.001	1.29	(1.15, 1.45)	<0.001
LMR	0.72	(0.65, 0.79)	<0.001	0.73	(0.66, 0.80)	<0.001	0.75	(0.66, 0.85)	<0.001
CAR	3.80	(3.29, 4.44)	<0.001	3.80	(3.28, 4.44)	<0.001	3.82	(3.18, 4.64)	<0.001
CALLY	0.51	(0.46, 0.57)	<0.001	0.51	(0.46, 0.57)	<0.001	0.56	(0.49, 0.64)	<0.001
CCR	4.64	(3.97, 5.47)	<0.001	4.68	(4.00, 5.52)	<0.001	4.84	(3.98, 5.96)	<0.001
CLR	2.28	(2.02, 2.58)	<0.001	2.28	(2.02, 2.58)	<0.001	2.12	(1.84, 2.46)	<0.001
RDW_Alb	1.86	(1.68, 2.06)	<0.001	1.85	(1.67, 2.06)	<0.001	1.74	(1.54, 1.99)	<0.001
SII	1.24	(1.14, 1.36)	<0.001	1.23	(1.12, 1.34)	<0.001	1.11	(0.98, 1.26)	0.091
NAR	1.63	(1.48, 1.79)	<0.001	1.61	(1.47, 1.78)	<0.001	1.44	(1.27, 1.64)	<0.001
SIRI	1.46	(1.33, 1.61)	<0.001	1.45	(1.31, 1.60)	<0.001	1.29	(1.15, 1.46)	<0.001

Continuous predictors included in the logistic regression models were standardized using z-score transformation. Accordingly, odds ratios (ORs) and 95% confidence intervals (CIs) represent the change in odds per one–standard deviation increase in the corresponding predictor. Logistic regression models were used to evaluate the association between inflammatory markers and the occurrence of moderately severe acute pancreatitis (MSAP) and severe acute pancreatitis (SAP). Model 1: Unadjusted. Model 2: Adjusted for gender and age. Model 3: Adjusted for gender, age, BMI, waist circumference, temperature, heart rate, respiratory rate, SBP, DBP, hypertension, diabetes, fatty liver, hyperlipidemia history, alcohol consumption, smoking, etiology, HCT, PLT, creatinine, BUN, sodium, potassium, chloride, PCT, HBP, lactate, PT, APTT, TT, and INR.

Inflammatory markers include NLR, PLR, MLR, LMR, CAR, CALLY, CCR, CLR, RDW/Alb, SII, NAR, and SIRI. Statistical significance was defined as *P* < 0.05.

OR, odds ratio; CI, confidence interval; MSAP, moderately severe acute pancreatitis; SAP, severe acute pancreatitis; BMI, body mass index; SBP, systolic blood pressure; DBP, diastolic blood pressure; HCT, hematocrit; PLT, platelet count; BUN, blood urea nitrogen; PCT, procalcitonin; HBP, heparin-binding protein; PT, prothrombin time; APTT, activated partial thromboplastin time; TT, thrombin time; INR, international normalized ratio; NLR, neutrophil-to-lymphocyte ratio; PLR, platelet-to-lymphocyte ratio; MLR, monocyte-to-lymphocyte ratio; LMR, lymphocyte-to-monocyte ratio; CAR, C-reactive protein-to-albumin ratio; CALLY, C-reactive protein-albumin-lymphocyte index; CCR, C-reactive protein-to-calcium ratio; CLR, C-reactive protein-to-lymphocyte ratio; RDW/Alb, red cell distribution width-to-albumin ratio; SII, systemic immune-inflammation index; NAR, neutrophil-to-albumin ratio; SIRI, systemic inflammation response index.

### ROC curve analysis

**Figure 2:**
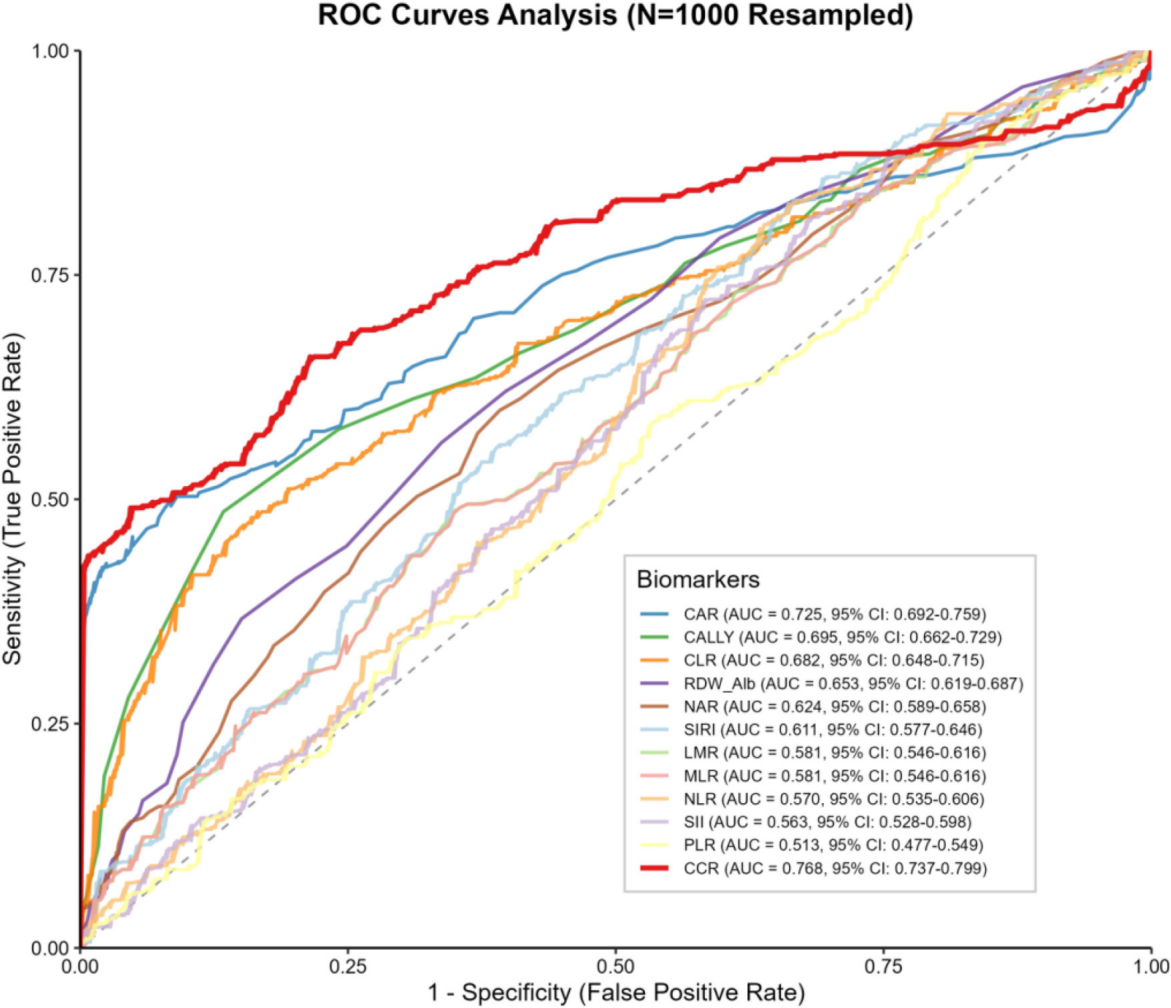
Receiver operating characteristic (ROC) curves for inflammatory markers in predicting the onset of moderately severe and severe acute pancreatitis (MSAP/SAP). MAP, mild acute pancreatitis; MSAP, moderately severe acute pancreatitis; SAP, severe acute pancreatitis; ROC, receiver operating characteristic; AUC, area under the curve; CI, confidence interval; NLR, neutrophil-to-lymphocyte ratio; PLR, platelet-to-lymphocyte ratio; MLR, monocyte-to-lymphocyte ratio; LMR, lymphocyte-to-monocyte ratio; CAR, C-reactive protein-to-albumin ratio; CALLY, C-reactive protein-albumin-lymphocyte index; CCR, C-reactive protein-to-calcium ratio; CLR, C-reactive protein-to-lymphocyte ratio; RDW/Alb, red cell distribution width-to-albumin ratio; SII, systemic immune-inflammation index; NAR, neutrophil-to-albumin ratio; SIRI, systemic inflammation response index.

ROC curve analysis showed that CCR had the highest AUC for predicting MSAP/SAP (AUC = 0.768, 95%CI: 0.737–0.799), indicating better discriminatory performance than the other indices (Figure 2). Additionally, RCS curve analysis indicated that, except for NAR, RDW/Alb, and MLR, all other inflammatory indices exhibited significant nonlinear relationships with the risk of MSAP/SAP (*P* < 0.05). After performing internal validation using bootstrap resampling, CCR remained the highest-performing inflammatory index, demonstrating the highest optimism-corrected AUC. Pairwise comparisons using the DeLong test confirmed that the AUC of CCR was significantly higher than those of the other indices (all *P* < 0.05).

Given the evaluation of multiple inflammatory indices across several models, including spline and threshold analyses, CCR was selected as the primary index for further in-depth analyses due to its numerically higher ROC-AUC in the main models.

### RCS analysis

**Figure 3:**
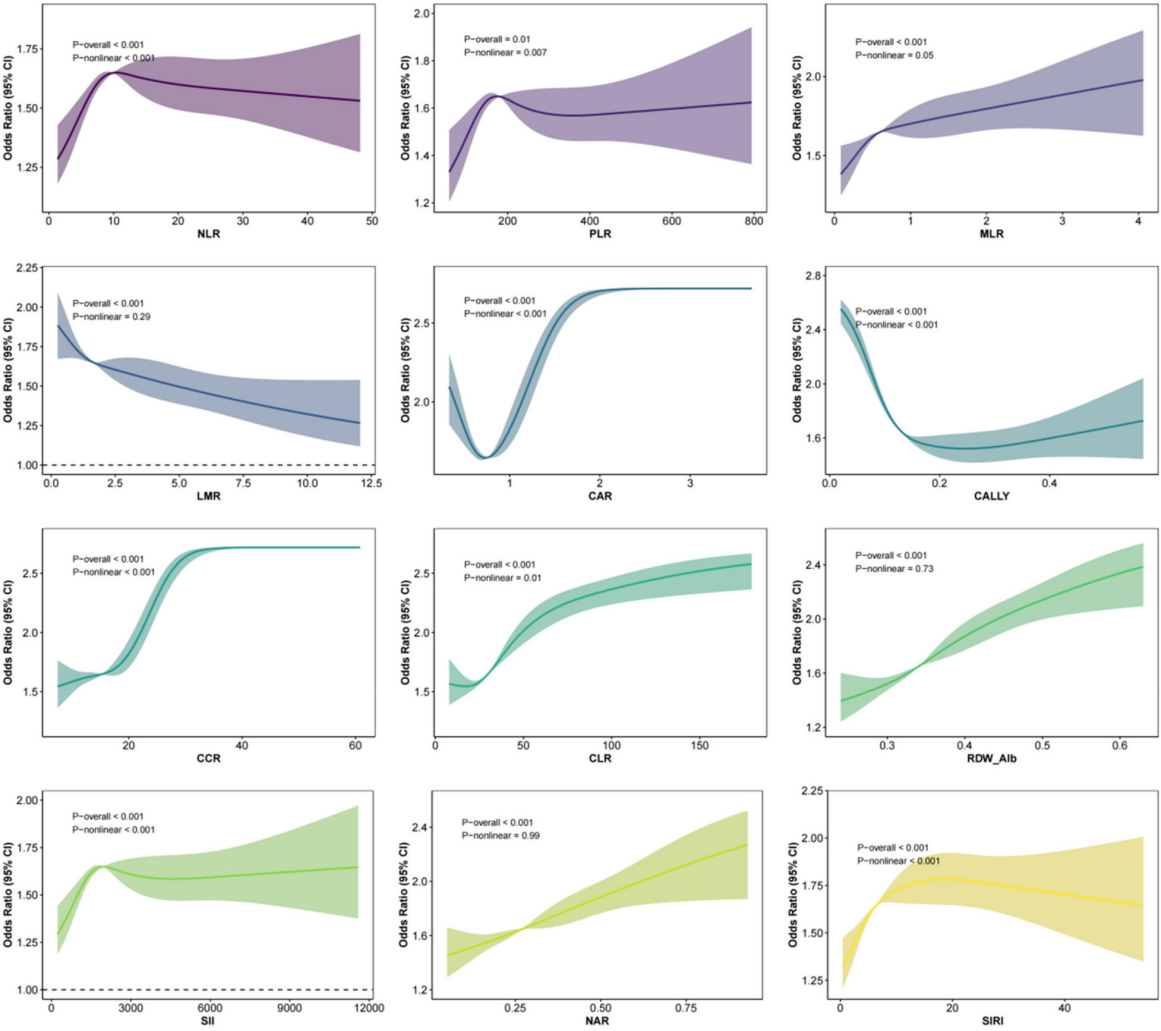
Restricted cubic spline (RCS) analysis of the nonlinear association between inflammatory markers and the risk of moderately severe and severe acute pancreatitis (MSAP/SAP). RCS models with four knots (located at the 5th, 35th, 65th, and 95th percentiles) were applied to explore the nonlinear dose–response relationship between each inflammatory marker and the occurrence of MSAP/SAP. All models were adjusted for the covariates in Model 3: gender, age, body mass index, waist circumference, temperature, heart rate, respiratory rate, systolic blood pressure, diastolic blood pressure, hypertension, diabetes, fatty liver, hyperlipidemia history, alcohol consumption, smoking, etiology, hematocrit, platelet count, creatinine, blood urea nitrogen, sodium, potassium, chloride, procalcitonin, heparin-binding protein, lactate, prothrombin time, activated partial thromboplastin time, thrombin time, and international normalized ratio. Solid lines represent the adjusted ORs, with 95% CIs indicated by shaded areas. The reference point (OR = 1) was set at the median value of each inflammatory marker. *P* values for overall association (P_overall) and nonlinearity (P_nonlinear) are presented in the table below the figure. RCS, restricted cubic spline; MSAP, moderately severe acute pancreatitis; SAP, severe acute pancreatitis; OR, odds ratio; CI, confidence interval; P_overall, *P* value for overall association; P_nonlinear, *P* value for nonlinearity; NLR, neutrophil-to-lymphocyte ratio; PLR, platelet-to-lymphocyte ratio; MLR, monocyte-to-lymphocyte ratio; LMR, lymphocyte-to-monocyte ratio; CAR, C-reactive protein-to-albumin ratio; CALLY, C-reactive protein-albumin-lymphocyte index; CCR, C-reactive protein-to-calcium ratio; CLR, C-reactive protein-to-lymphocyte ratio; RDW/Alb, red cell distribution width-to-albumin ratio.

RCS analysis demonstrated that NLR, PLR, MLR, CAR, CALLY, CCR, CLR, SII, and SIRI were significantly associated with the risk of MSAP/SAP, showing pronounced nonlinear dose–response relationships. In contrast, LMR, RDW/Alb, and NAR exhibited significant overall associations but without meaningful nonlinear trends. The detailed dose–response curves are presented in Figure 3.

### Threshold effect analysis

Further threshold effect analysis revealed a significant nonlinear association between CCR and the risk of MSAP/SAP (*P* < 0.001). The conventional logistic regression model suggested that for every one-unit increase in CCR, the risk of MSAP/SAP increased by 15.7% (OR = 1.157, 95%CI: 1.14–1.176, *P* < 0.001). The segmented logistic regression model an inflection point (change point) at CCR = 15. When CCR < 15, there was no significant association with MSAP/SAP risk (OR = 1.015, 95%CI: 0.965–1.068, *P* = 0.558); however, when CCR ≥ 15, the risk of MSAP/SAP significantly increased with increasing CCR (OR = 1.212, 95%CI: 1.182–1.245, *P* < 0.001). The likelihood ratio test further supported that the segmented logistic regression model provided a better fit than the conventional logistic regression model (*P* < 0.001).

To further evaluate the potential clinical triage utility of CCR, diagnostic performance metrics were calculated at the optimal cutoff value (CCR = 16.835). At this threshold, CCR demonstrated a sensitivity of 0.659 and a specificity of 0.785. The positive predictive value (PPV) was 0.730, and the negative predictive value (NPV) was 0.723. In addition, the positive likelihood ratio (LR+) was 3.069, and the negative likelihood ratio (LR−) was 0.434, indicating a moderate ability of CCR to discriminate patients at higher risk of MSAP/SAP at admission. The threshold effect analysis results are presented in Table 3.

**Table 3:** Threshold effect analysis results for CCR.

Analysis Method	Effect Size (95% CI), *P* value
Model 1: Conventional logistic regression	1.157 (1.14–1.176), *P* < 0.001
Model 2: Segmented logistic regression	—
Inflection point	15
CCR <15	1.015 (0.965–1.068), *P* = 0.558
CCR >15	1.212 (1.182–1.245), *P* < 0.001
Likelihood ratio test *P* value	*P* < 0.001

MAP, mild acute pancreatitis; MSAP, moderately severe acute pancreatitis; SAP, severe acute pancreatitis; CCR, C-reactive protein-to-calcium ratio.

## Discussion

This study employed logistic regression analysis to evaluate the association between multiple inflammation-related markers and the severity of AP. After full adjustment for covariates, NLR, PLR, and SII showed no significant association; however, MLR, LMR, CAR, CALLY, CCR, CLR, RDW/Alb, NAR, and SIRI were significantly associated with AP severity. ROC curve analysis demonstrated that CCR had the highest observed efficacy for AP severity, showed a greater AUC than other indices RCS analysis further revealed significant nonlinear associations between most markers (except NAR, RDW/Alb, and MLR) and the risk of MSAP/SAP. Threshold effect analysis of CCR confirmed its nonlinear relationship with MSAP/SAP risk, with an inflection point at 15: CCR values below 15 were not associated with increased risk, whereas values above 15 were linked to a significantly higher risk. Likelihood ratio testing supported the superiority of the two-piecewise model over a linear model.

Compared with previous research, several studies have reported associations between inflammatory markers such as SII, CLR, and NLR and AP severity. For example, Zhang et al. (2021) found SII to be a potential early predictor of AP severity (14). Li et al. reported that CLR was positively correlated with the risk of severe AP, noting that excessive IL-6 release in severe cases promotes CRP deposition at inflammatory sites, amplifying the pro-inflammatory response (11). Jain et al. observed that RDW, NLR, and LMR were comparable to established scoring systems in predicting AP-related mortality and inflammatory severity (25). Dao et al. suggested that SIRI, when combined with BISAP, can predict SAP severity (30). Jeon et al. highlighted the predictive value of elevated NLR for SAP severity and organ failure (31). In contrast, in our fully adjusted multivariable model, SII, CLR, and NLR were not significantly associated with AP severity. This discrepancy may be explained by differences in study populations or by the adjustment for acute infection markers such as procalcitonin in our analysis, which excluded confounding hematologic responses to infection. Consequently, these markers did not demonstrate stronger associations compared to CRP or its composite indices.

Prior research has demonstrated that CAR is a reliable predictor of AP severity (18), and Kaplan et al. further reported that elevated CAR is associated with an increased risk of mortality (32). Uğurlu et al. suggested that admission CAR could serve as a prognostic marker for adverse outcomes in AP (33). However, most prior studies did not directly compare composite indices with single CRP values. Ahmad R, for example, indicated that elevated CRP within 48 h may reflect complications unrelated to AP, limiting its reliability in predicting severe disease (34). In contrast, our study found CCR to be relatively higher to CRP alone (higher AUC) and established a precise threshold effect (CCR ≥15), thereby addressing gaps in prior research.

Previous findings have highlighted the roles of serum calcium and CRP in AP. Pokharel et al. reported that albumin-corrected calcium within 24 h was predictive of AP severity (35). Chhabra et al. emphasized that hypocalcemia consistently influences AP severity and mortality, regardless of etiology (36). Li et al. linked high admission CRP to increased SAP risk (37), while Cardoso et al. confirmed the predictive accuracy of CRP within 48 h of admission (38). As a composite marker, CCR combines CRP and serum calcium and demonstrated significant association with AP severity. The underlying mechanisms can be explained by CRP being a systemic response to pro-inflammatory cytokines such as IL-6, which are elevated in AP (16). In SAP, enzyme release intensifies inflammation, leading to fat necrosis and tissue damage, further depleting calcium through saponification (39). This dual impact of inflammation and calcium consumption may drive disease progression toward more severe clinical types (13). However, Bilgili et al. argued that hypocalcemia can occur as a general response to acute inflammation in various conditions, including trauma, malignancy, and infection (40). Chen et al. reported that elevated CCR remained significantly associated with MSAP/SAP risk after adjustment for confounders (13). The strength of CCR lies in its ability to capture the dynamic balance overlooked by single biomarkers, explaining its nonlinear threshold effect: CCR <15 may indicate a controlled inflammatory state, whereas CCR ≥15 may trigger a vicious cycle.

The clinical significance of this study lies in its systematic evaluation of CCR and other composite inflammatory markers for predicting acute pancreatitis (AP) severity using a large retrospective cohort dataset. Although CCR was not prespecified *a priori* as the sole primary biomarker, it was prioritized for further nonlinear and threshold analyses due to its consistent performance across adjusted models and internal validation, with all other indices considered exploratory. The identified threshold effect (CCR ≥ 15) at admission provides a clinically meaningful early warning signal that may assist in early risk stratification and triage, rather than serving as a standalone predictive model. Given the ease of measurement and wide availability of serum calcium and C-reactive protein, CCR represents a practical and accessible tool, particularly in resource-limited settings. When CCR is ≥15, clinicians may consider closer monitoring or timely initiation of anti-inflammatory interventions. Moreover, the observed nonlinear relationship between CCR and AP severity highlights the importance of accounting for complex biomarker dynamics, which may support more personalized management strategies, ultimately reducing AP-related morbidity, mortality, and healthcare burden while improving patient outcomes.

The strengths of this study include comprehensive multivariable adjustment to minimize confounding, the use of RCS and threshold analyses to uncover nonlinear associations, and a large sample size that enhanced statistical power. Nevertheless, certain limitations should be acknowledged: this study has several limitations. First, it is based on a single-center, retrospective cohort, which may limit the generalizability of our findings to other populations or settings. Second, although we adjusted for a wide range of potential confounders, residual confounding from unmeasured factors, such as dynamic clinical changes or additional biomarkers, may still influence the results. Third, the study did not incorporate real-time dynamic biomarkers or continuous monitoring, which could provide a more nuanced understanding of the patient's condition over time. Lastly, while our analysis focused on identifying clinically meaningful cutoffs, the potential for overadjustment in the models exists, particularly in the more robust etiologic models.

Several limitations of this study should be acknowledged. First, this was a single-center retrospective cohort study, and selection bias cannot be entirely excluded, which may limit the generalizability of the findings. Second, variables with substantial non-random missingness were excluded from the primary analyses to avoid inappropriate imputation, which may have influenced effect estimates. In addition, reliance on self-reported medical history may have introduced recall bias. Finally, dynamic changes in inflammatory biomarkers during hospitalization were not assessed, and future prospective multicenter studies with predefined sampling protocols are warranted to validate our findings.

In conclusion, this study provides new insights into AP management, highlighting CCR as a potential biomarker. Future multicenter prospective studies are needed to validate its applicability across diverse populations and to explore its relationship with treatment response, with the ultimate goal of optimizing clinical management strategies and improving patient outcomes.

## Data Availability

The datasets used and/or analyzed during the current study are available from the corresponding author on reasonable request. Requests to access these datasets should be directed to Qiaojie Bi, 19246316650@163.com.
