# Supplementary material for: The association between inflammatory indices and acute pancreatitis severity: a retrospective cohort study
Source: Front Surg. 2026 Feb 26;13:1764029. doi: 10.3389/fsurg.2026.1764029 (PMC12979465; doi:10.3389/fsurg.2026.1764029)
Supplement: Supplementary file 1 [file Table1.docx]

# Supplementary Table 1. Multicollinearity Assessment of Model Covariates

| **Variable** | **VIF** |
| --- | --- |
| Systolic Blood Pressure (mmHg) | 1.236 |
| Diastolic Blood Pressure (mmHg) | 1.206 |
| Respiratory Rate (bpm) | 1.145 |
| Heart Rate (bpm) | 1.129 |
| Body Mass Index (kg/m²) | 1.095 |
| Age (years) | 1.081 |
| Diabetes Mellitus | 1.063 |
| Fatty Liver Disease | 1.046 |
| Serum Lactate (mmol/L) | 1.043 |
| Hyperlipidemia History | 1.041 |
| Gender | 1.039 |
| Heparin-binding Protein (pg/mL) | 1.038 |
| Platelet Count (×10⁹/L) | 1.033 |
| Procalcitonin (ng/mL) | 1.033 |
| Hematocrit (%) | 1.028 |
| Serum Sodium (mmol/L) | 1.026 |
| Hypertension | 1.024 |
| Serum Chloride (mmol/L) | 1.016 |
| Body Temperature (°C) | 1.016 |
| Smoking Status | 1.016 |
| Waist Circumference (cm) | 1.013 |
| Activated Partial Thromboplastin Time (s) | 1.013 |
| Alcohol Consumption | 1.013 |
| Prothrombin Time (s) | 1.013 |
| Thrombin Time (s) | 1.010 |
| Etiology | 1.009 |
| International Normalized Ratio | 1.008 |
| Blood Urea Nitrogen (mmol/L) | 1.008 |
| Serum Potassium (mmol/L) | 1.008 |
| Serum Creatinine (μmol/L) | 1.007 |

Note. VIF, Variance Inflation Factor. Values represent GVIF^(1/(2×df)) for categorical variables and traditional VIF for continuous variables. All covariates demonstrated VIF values < 5, indicating no concerning multicollinearity.
